# Lupus of the Mouth and Pharynx

**Published:** 1877-04

**Authors:** G. Homolle


### ARTICLE X.

### *Lupus of the Mouth and Pharynx.*

In the *Central'latt f. d. Med. Wissensch.*, No. 55, 1875, we find an extract from a work by G. Homolle, entitled "*Des Scrophulieds Graves de la Muquese Bucco pharyngilune*," from which we glean the following:

Lupus of the buccal or pharyngeal cavity generally manifests itself as a complication of lupus of the external skin. Only in rare instances does it manifest itself primarily if the cuts be free. In the former instance the mucous membrane presents the following forms of disease: livid erythema, granulations, an hypertrophic and rarely an atrophic appearance, ulcerations, perforations, and lastly the exceedingly rare transformation into an epithelioma—appearances which are markedly influenced by the seat of the affection. The depression, pale or brownish hue of the remaining cicatrices are not characteristic. Stellate cicatrices of the pharnyx, as well as the less frequent adhesions between the same and the soft palate, are also observed. The peculiar indolence of the symptoms, even in the more destructive onslaughts of the disease, may be considered a functional symptom, in which case the sensibility of the parts is markedly diminished.

Primary lupus does not differ from secondary as far as the elementary lesions are concerned. Two forms may be mentioned: primary lupus, when the affected parts become atrophic, and the primative ulcerative scrofulides, if the same undergo destruction. Primary lupus generally presents conglomerate knots, sometimes of a hypertrophic type; very rarely are isolated knots noticed. The ulcerating scrofulides begin with a purulent discharge, and soon give rise to deformities. "Symptomatically, these diseases do not generally proceed in as indolent a manner as secondary lupus."

Sometimes we notice as complications glandular enlargements, ottorrhœa, keratitis, conjunctivitis, and very rarely œddma glottidis. As far as the general condition goes, four clinical types may be observed, varying with the subject: 1. If they be healthy and well nourished. 2. If they although of a healthy appearance, reveal some traces of scrofulous disease. 3. A decidedly strumous habit. 4. If they be affected by congenital syphilis (in young subjects). The patients are generally from ten to twenty years of age.

The author is very decided in his opinion that lupus is an undoubted scrofulous disease, and calls all ulcerative processes scrofulides.

The diagnosis of primary lupus of the mucous membrane, particularly of the ulcerative forms, is a very difficult one. Only after a very careful examination, an exact and truthful history, and a due observation of the train of syphilis, will we be able to exclude syphilis. In consequence of the beneficial effects afforded by anti-syphilitic measures, we must be the more careful in our diagnosis.

The prognosis is generally dubious, particularly in regard to a complete cure. Relapses are of frequent occurrence.

The first indication in treatment is to improve the general health. The administration of cod liver oil and iodine are the most fruitful. Locally, tincture of iodine, chromic acid, chloride of zinc, nitrate of silver, etc., and during severe pains iodoform in glycerine should be applied.—*Louisville Med News.*

